# Enhanced glucose uptake promotes the survival of hepatocellular carcinoma cells following incomplete radiofrequency ablation

**DOI:** 10.1080/23723556.2026.2715876

**Published:** 2026-08-25

**Authors:** Xiaofeng Wang, Ting Li, Chao Peng

**Affiliations:** a Department of Emergency Medicine, Gansu Provincial Maternity and Child-Care Hospital/Gansu Provincial Central Hospital, Lanzhou, Gansu, People's Republic of China; b Department of Anesthesiology, Gansu Provincial People's Hospital, Lanzhou, Gansu, People's Republic of China; c Department of Gastric Cancer, Sun Yat-sen University Cancer Center Gansu Hospital, Gansu, Lanzhou, People's Republic of China; d Department of Gastric Surgery, Gansu Provincial Cancer Hospital, Lanzhou, Gansu, People's Republic of China

**Keywords:** Incomplete radiofrequency ablation (iRFA), hepatocellular carcinoma (HCC), GLUT4, glucose uptake, reactive oxygen species (ROS)

## Abstract

Incomplete radiofrequency ablation (iRFA) exposes residual hepatocellular carcinoma (HCC) cells to sublethal thermal stress, which triggers metabolic adaptations that support cell survival. In this study, we demonstrate that iRFA significantly enhances glucose uptake in HCC cells, contributing to the suppression of intracellular reactive oxygen species (ROS) and promoting cell viability. Mechanistically, this effect is mediated by the translocation of GLUT4 from the cytoplasm to the plasma membrane, rather than changes in total GLUT4 expression. GLUT4 redistribution under thermal stress facilitates rapid glucose uptake, providing energy and biosynthetic intermediates necessary for survival under adverse microenvironmental conditions. Inhibition of GLUT4-mediated glucose uptake increases ROS accumulation and reduces cell survival, highlighting its critical role in redox homeostasis. Collectively, our findings reveal an iRFA–GLUT4–glucose uptake axis as a key metabolic adaptation pathway that enables residual tumor cells to survive sublethal thermal stress, suggesting that targeting GLUT4 dynamics may represent a potential therapeutic strategy to improve the efficacy of iRFA and prevent tumor recurrence.

## Introduction

Hepatocellular carcinoma (HCC) remains one of the most prevalent malignancies worldwide and a leading cause of cancer-related mortality, representing a substantial global health burden.^1^ For patients with early-stage disease, curative treatments include surgical resection, liver transplantation, and local ablative therapies.^2^ Among these, radiofrequency ablation (RFA), a minimally invasive and repeatable local modality, is widely applied in early-stage HCC and in patients unsuitable for surgery. Nevertheless, the high recurrence rate following incomplete RFA (iRFA) poses a major clinical challenge.^3^ ^,^ ^4^ Incomplete ablation has been identified as an independent risk factor negatively impacting local tumor control and recurrence-free survival. Importantly, residual tumor cells after iRFA are not dormant but often exhibit enhanced proliferative, invasive, and metastatic capacities.^5^ ^,^ ^6^

Metabolic reprogramming, particularly the aberrant activation of glucose metabolism, is recognized as a critical driver of tumor initiation, progression, and therapeutic resistance. Tumor cells enhance glucose uptake and glycolytic flux to meet the energetic and biosynthetic demands of rapid proliferation, apoptosis resistance, and invasion.^7^ This is typically accompanied by upregulation of glucose transporters and key glycolytic enzymes and contributes to the maintenance of malignant phenotypes via modulation of redox homeostasis, epigenetic modifications, and multiple signaling pathways.^8^

As a form of sublethal thermal injury, iRFA exposes residual cells to transient heat stress, hypoxia, and an inflammatory microenvironment—factors known to induce glycolytic activation. Studies have shown that heat stress enhances glucose uptake and triggers metabolic reprogramming.^9^ ^,^ ^10^ Short-term hyperthermia reduces cellular energy status, activating the AMPK energy-sensing pathway and promoting insulin-independent glucose transport.^11^ Prolonged heat stress further upregulates glucose transporters and multiple glycolytic enzymes, increasing cellular glucose utilization.^12^ ^,^ ^13^ Nevertheless, how glucose uptake is dynamically regulated and how it contributes to the survival of HCC cells after iRFA remain largely unknown. Therefore, this study aimed to investigate how iRFA alters glucose uptake in residual tumor cells and to elucidate the underlying molecular mechanisms supporting cell survival.

## Materials and methods

### Cell viability assessment by CCK-8 assay

Cell viability was assessed using the Cell Counting Kit-8 (CCK-8, Beyotime, China) assay according to the manufacturer’s instructions. Briefly, hepatocellular carcinoma cells were seeded into 96-well plates at an appropriate density and subjected to indicated conditions. At the specified time points after treatment, CCK-8 reagent was added to each well at a ratio of 10 μL per 100 μL of culture medium. Cells were then incubated at 37 °C for 1–2 h, and the absorbance at 450 nm was measured using a microplate reader. Cell viability was calculated as a percentage relative to the corresponding control group. Each experiment was performed in triplicate and repeated three times independently.

### Annexin V-FITC/PI flow cytometric analysis

Cell death was evaluated using Annexin V-FITC/propidium iodide (PI) staining(ThermoFisher, USA) according to the manufacturer’s instructions. Briefly, cells were harvested at the indicated time points after treatment, washed twice with cold PBS, and resuspended in binding buffer. Cells were then stained with Annexin V-FITC and PI for 15 min at room temperature in the dark. Samples were analyzed by flow cytometry, and the proportions of Annexin V/PI-positive populations were quantified. The total Annexin V-positive and/or PI-positive populations were used to evaluate cell death after iRFA treatment.

### Measurement of intracellular reactive oxygen species (ROS) by DCFH-DA staining

Intracellular ROS levels were measured using the fluorescent probe 2′,7′-dichlorofluorescein diacetate (DCFH-DA, MCE, USA). Briefly, cells were collected and washed twice with phosphate-buffered saline (PBS), then incubated with DCFH-DA (final concentration, 10 µM) at 37 °C for 30 min in the dark. After incubation, cells were washed three times with PBS to remove excess probe and resuspended in PBS for analysis. Fluorescence intensity was detected by flow cytometry using the FITC channel. The mean fluorescence intensity (MFI) was quantified to evaluate intracellular ROS levels.

### Measurement of glucose uptake by 2-NBDG staining

Cellular glucose uptake was assessed using the fluorescent glucose analog 2-(*N*-(7-nitrobenz-2-oxa-1,3-diazol-4-yl)amino)-2-deoxyglucose(2-NBDG, MCE, USA). Cells were harvested, washed with PBS, and incubated with 2-NBDG (final concentration, 100 µM) in glucose-free medium at 37 °C for 30 min in the dark. Following incubation, cells were washed twice with cold PBS to terminate glucose uptake. The fluorescence intensity of 2-NBDG was measured by flow cytometry using the FITC channel. Glucose uptake was quantified as the MFI of 2-NBDG-positive cells.

### Measurement of glucose concentration in culture medium

Glucose concentration in the culture medium was determined using a glucose assay kit (Beyotime, China) based on the glucose oxidase/peroxidase (GOD/POD) colorimetric method, according to the manufacturer’s instructions. Briefly, culture supernatants were collected at the indicated time points and centrifuged to remove cellular debris. Samples were then incubated with the working reagent at 37 °C for the recommended duration, allowing glucose to be oxidized by glucose oxidase and subsequently react with peroxidase to produce a colored product. The absorbance was measured at 505 nm using a microplate reader. Glucose concentrations were calculated based on a standard curve generated using known glucose standards and normalized to cell number where indicated. All measurements were performed in triplicate.

### Western blot analysis

Cells were lysed in RIPA buffer on ice for 30 min. Lysates were clarified by centrifugation at 12,000 × g for 15 min at 4 °C, and the supernatants were collected. Protein concentrations were determined using a BCA protein assay kit (Beyotime, China). Equal amounts of protein were separated by SDS-PAGE and transferred onto polyvinylidene difluoride (PVDF) membranes. Membranes were blocked with bovine serum albumin (BSA) in Tris-buffered saline containing 0.1% Tween-20 (TBST) for 1 h at room temperature and then incubated with primary antibodies against overnight at 4 °C. After washing with TBST, membranes were incubated with appropriate HRP-conjugated secondary antibodies for 1 h at room temperature. Protein bands were visualized using an enhanced chemiluminescence (ECL) detection systemand imaged with a chemiluminescence imaging system. Band intensities were quantified using ImageJ software, and target protein expression levels were normalized to GAPDH/ATP1A.

### Plasma membrane and cytoplasmic protein fractionation

Plasma membrane and cytoplasmic proteins were isolated using a Membrane Protein Extraction Kit (Beyotime, China) according to the manufacturer's instructions. Briefly, cells were harvested and washed twice with ice-cold PBS. The cell pellet was resuspended in membrane protein extraction buffer supplemented with protease and phosphatase inhibitors and incubated on ice for 15 min. After homogenization, samples were centrifuged at 700 × g for 10 min at 4 °C to remove nuclei and cell debris. The resulting supernatant was subsequently centrifuged at 14,000 × g for 30 min at 4 °C. The supernatant containing cytoplasmic proteins was collected and stored as the cytosolic fraction. The pellet containing membrane proteins was resuspended in membrane protein solubilization buffer and incubated on ice for 30 min with intermittent vortexing. Following centrifugation at 14,000 × g for 15 min, the supernatant was collected as the membrane protein fraction.

Protein concentrations were determined using a BCA Protein Assay Kit. Equal amounts of protein from each fraction were subjected to Western blot analysis. Na⁺/K⁺-ATPase was used as a plasma membrane marker, whereas GAPDH served as a cytoplasmic marker to verify the purity of the isolated fractions. GLUT4 expression in membrane and cytoplasmic fractions was analyzed by Western blotting to evaluate GLUT4 membrane translocation.

### Immunofluorescence staining

Cells were seeded on sterile glass coverslips and cultured under the indicated conditions. After treatment, cells were washed with PBS and fixed with 4% paraformaldehyde for 15 min at room temperature. Cells were then permeabilized with 0.1% Triton X-100 in PBS for 10 min and blocked with 5% BSA for 1 h at room temperature. Cells were incubated with a primary antibody against GLUT4 (Abcam, USA) overnight at 4 °C. After washing with PBS, cells were incubated with an appropriate fluorophore-conjugated secondary antibody for 1 h at room temperature in the dark. Nuclei were counterstained with DAPI for 5 min. Fluorescence images were captured using a fluorescence or confocal microscope.

### Quantitative real-time PCR

Total RNA was extracted from cells using Trizol reagent according to the manufacturer’s instructions. Complementary DNA (cDNA) was synthesized from 1 µg of total RNA using a reverse transcription kit. Quantitative real-time PCR was performed using SYBR Green PCR Master Mix on a real-time PCR system. The amplification conditions were as follows: initial denaturation at 95 °C for 5 min, followed by 40 cycles of denaturation at 95 °C for 10 s and annealing/extension at 60 °C for 30 s. Melt curve analysis was performed to confirm the specificity of amplification. Gene expression levels were normalized to an internal control gene GAPDH, and relative mRNA expression was calculated using the 2^−ΔΔCt method. The primers employed were: GAPDH F5’-TGACAACAGCCTCAAGAT-3’, R5’-GAGTCCTTCCACGATACC-3’, GLUT1 F5’-GGCCAAGAGTGTGCTAAAGAA-3’, R5’-ACAGCGTTGATGCCAGACAG-3’, GLUT3 F:5’-GCGTGCTAGCTCGAGGCCCGCCACCATGCCCGGCT-3’, R5’-CGGAATGCCAAGCTTTGTGATCTCTCGGAATTCTG-3’, GLUT4 F 5’-GGGCTGAGACAGGGACCATAAC-3’, R5’-CATGAGCAATGGCATCC CAGAA-3’.

### siRNA transfection

Small interfering RNAs (siRNAs) targeting GLUT4 and a non-targeting negative control siRNA were purchased from Genepharma (China). Cells were seeded in culture plates and transfected at 60–70% confluence using Lipofectamine RNAiMAX (ThermoFisher, USA) according to the manufacturer’s instructions. Briefly, siRNAs and transfection reagent were separately diluted in Opti-MEM (Gibco, USA), mixed gently, and incubated for 10–15 min at room temperature to allow complex formation. The complexes were then added to the cells at a final siRNA concentration of 50 nM. After 6 h of incubation, the medium was replaced with fresh complete culture medium. iRFA treatment was performed 24–48 h after transfection when GLUT4 knockdown efficiency was confirmed. Gene silencing efficiency was confirmed by quantitative real-time PCR and Western blot analysis 24–48 h after transfection. The sequences used were as follows: siCtrl F 5’- UUCUCCGAACGUGUCACGU-3’, R5’- ACGUGACACGUUCGGAGAA-3’.siGLUT4-1 F 5’- GCUUCUUCUACCUCUUCAA-3’, R5’- UUGAAGAGGUAGAAGAAGC-3’.siGLUT4-2 F 5’- CCAUCUUCUUCAUCGUUAU-3’, R5’- AUAACGAUGAAGAAGAUGG-3’.

### In vitro iRFA model

An in vitro iRFA model was established with minor modifications from previously reported sublethal thermal stress protocols.^14^ This model was designed to simulate the biological responses of residual HCC cells located in the peripheral transition zone following incomplete ablation, where tumor cells are exposed to sublethal thermal stress rather than the lethal temperatures causing complete coagulative necrosis.

HepG2 and HuH7 cells cells were plated in 6-well plates and cultured until reaching approximately 70–80% confluence. Prior to heat treatment, the culture medium was preheated to 46 °C and added to the wells. The culture plates were then sterilely sealed with Parafilm M to prevent evaporation and water bath contamination during immersion. The sealed plates were completely submerged in a temperature-controlled water bath maintained at 46.0 ± 0.1 °C for 15 min. The water bath temperature was continuously monitored throughout the treatment period to ensure stable thermal exposure. Immediately after heat exposure, the sealing film was removed under sterile conditions, and the culture medium was replaced with fresh complete medium pre-warmed to 37 °C. Cells were then returned to a standard humidified incubator (37 °C, 5% CO₂) for recovery and subsequent downstream analyzes at the indicated time points.

### Measurement of lactate

Lactate concentrations were determined using commercially available lactate assay kits according to the manufacturer’s instructions (Solarbio, China). Briefly, cell culture supernatants were collected, and lactate levels were measured using a microplate reader at the specified wavelength. Lactate concentrations were calculated based on a standard curve and normalized to protein concentration where indicated.

### Statistical analysis

All quantitative data are expressed as the mean ± standard error (SE). Statistical evaluation was conducted with SPSS Statistics version 25 (IBM, USA). For comparisons between two experimental groups, an unpaired Student’s t-test was applied. For analyzes involving three or more groups, one-way analysis of variance (ANOVA) followed by Tukey’s multiple comparisons test was used. Differences with a *p*-value less than 0.05 were considered statistically significant. Graphical representations were created using GraphPad Prism 8.0 software.

## Results

### iRFA enhances glucose uptake in HCC cells

**Figure 1.**
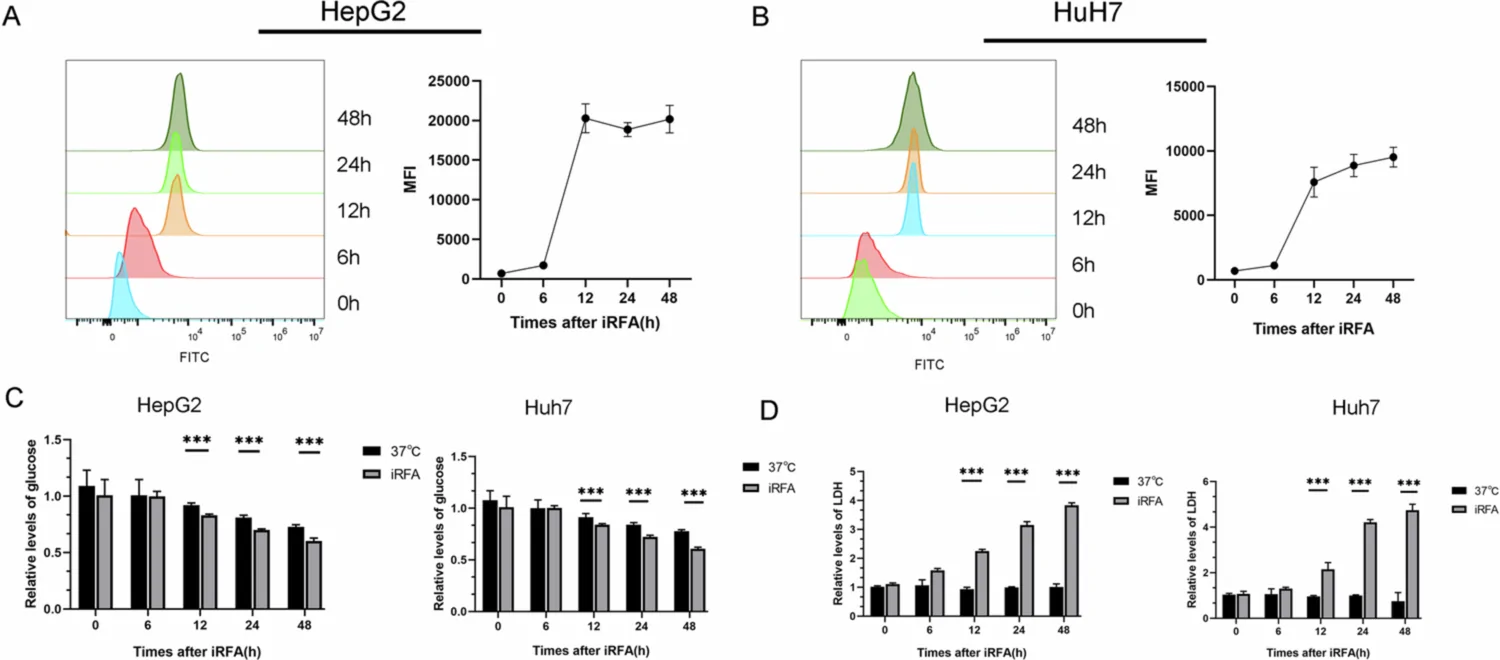
Insufficient thermal ablation enhances glucose uptake in hepatocellular carcinoma cells. (A, B) Cellular glucose uptake was evaluated by flow cytometric analysis of 2-NBDG uptake in HepG2 and HuH7 cells at the indicated time points after insufficient thermal ablation (iRFA). Representative histograms (A) and quantitative analysis (B) show a significant increase in glucose uptake following heat stress. (C) Glucose concentration in the culture medium was measured using a GOD/POD-based colorimetric assay at different time points after iRFA. (D) Lactate levels in the culture medium were determined to assess glycolytic activity following heat treatment. *p < 0.05; **p < 0.01; ***p < 0.001. Data are presented as mean ± SD from at least three independent experiments.

To determine whether iRFA affects glucose metabolism in HCC cells, glucose uptake was evaluated using the fluorescent glucose analog 2-NBDG at different time points following iRFA treatment. Flow cytometric analysis revealed a significant increase in glucose uptake after iRFA in both HepG2 and HuH7 cells. Compared with the baseline level, 2-NBDG uptake increased markedly between 6 and 12 h after iRFA and remained elevated for at least 48 h (Figure 1A and 1B), indicating a sustained enhancement of cellular glucose uptake following sublethal thermal stress.

To further assess metabolic alterations induced by iRFA, glucose consumption and lactate production were measured. Consistent with the increased 2-NBDG uptake, the concentration of glucose remaining in the culture medium was significantly reduced at 12 h after iRFA compared with control cells (Figure 1C), suggesting increased glucose utilization by HCC cells. In parallel, lactate accumulation was significantly elevated from 6 to 12 hours post-iRFA and remained sustained thereafter (Figure 1D), indicating enhanced glycolytic activity.

Collectively, these findings demonstrate that iRFA induces a sustained increase in glucose uptake and glycolytic metabolism in HCC cells, suggesting that surviving HCC cells undergo metabolic adaptation in response to sublethal thermal stress.

### Extracellular glucose availability promotes HCC cell survival after iRFA

**Figure 2.**
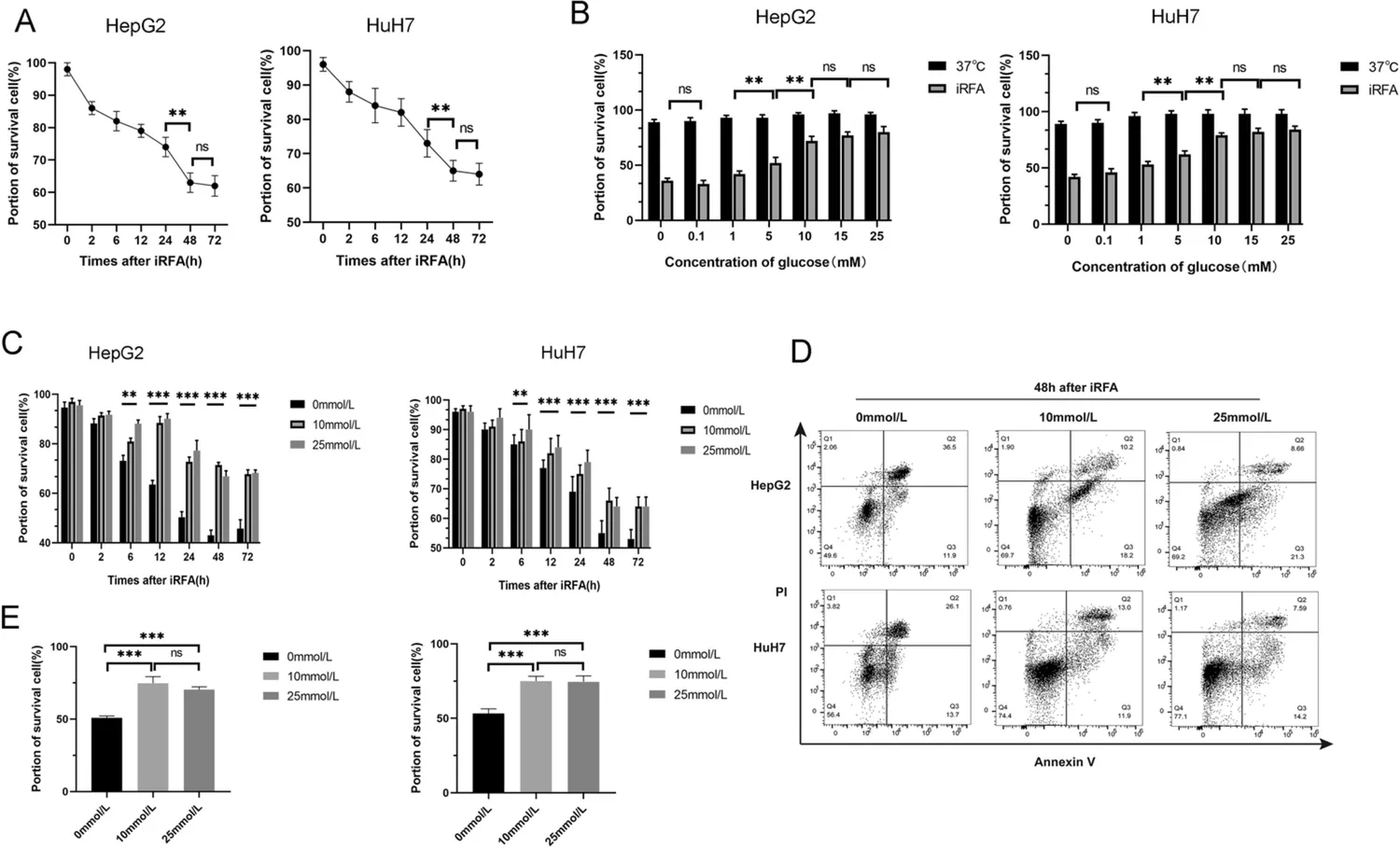
Extracellular glucose concentration correlates with HCC cell survival after iRFA. (A) Apoptosis of HepG2 and Huh7 cells was analyzed at the indicated time points following insufficient thermal ablation (iRFA). (B) Cell survival of HCC cells cultured in media containing different glucose concentrations (0, 0.1, 1, 5, 10, 15 and 25 mM) was assessed 48 h after iRFA. (C) Time-course analysis of cell death in HCC cells cultured under different extracellular glucose concentrations during the 0–72 h period following iRFA. (D) Cell survival cultured in media containing different glucose concentrations (0, 10 and 25 mM) was quantified using Annexin V-FITC/PI staining and flow cytometry at 48 h after iRFA. (E) The statistical results of D in three independent experiments. *p < 0.05; **p < 0.01; ***p < 0.001. Data are presented as mean ± SD from three independent experiments.

To establish the optimal time window for evaluating cellular responses after sublethal thermal stress, time-course cell viability was dynamically assessed using the CCK-8 assay at multiple time points (0, 2, 6, 12, 24, 48, and 72 h) following 46 °C thermal exposure. As shown in Figure 2A, cell viability progressively decreased after iRFA treatment and reached a relatively stable level at 48 h compared with sham controls. Therefore, 48 h after iRFA was selected as the representative time point for subsequent experiments investigating the impact of extracellular glucose availability on residual HCC cell survival.

To determine whether extracellular glucose availability influences cell survival under thermal stress conditions, HepG2 and HuH7 cells were cultured in media containing different glucose concentrations (0, 0.1, 1, 5, 10, 15 and 25 mM) immediately after iRFA exposure. At 48 h after iRFA, CCK-8 analysis demonstrated a glucose-dependent improvement in cell viability. Compared with glucose-deprived conditions (0 mM), supplementation with 5 mM glucose significantly restored cell viability, while higher glucose concentrations (≥10 mM) provided a more pronounced protective effect (Figure 2B). Consistently, longitudinal analysis over 0–72 h after iRFA showed that cells cultured under moderate (10 mM) or high (25 mM) glucose conditions maintained significantly higher viability compared with glucose-starved cells (Figure 2C).

To further determine whether the reduced viability under glucose deprivation was associated with increased cell death, Annexin V-FITC/PI flow cytometric analysis was performed at 48 h after iRFA under representative glucose conditions (0, 10, and 25 mM). Consistent with the CCK-8 results, glucose deprivation markedly increased the proportion of Annexin V/PI-positive cells in both HepG2 and HuH7 cells, whereas supplementation with 10 mM or 25 mM glucose significantly reduced iRFA-induced cell death (Figure 2D and 2E).

Collectively, these findings demonstrate that extracellular glucose availability is critical for maintaining the survival of HCC cells following sublethal thermal stress and suggest that metabolic adaptation through enhanced glucose utilization may represent an important survival mechanism after incomplete ablation.

### Glucose availability influences ROS homeostasis following iRFA

Intracellular ROS are important regulators of cellular stress responses and can contribute to cell injury and death when excessively accumulated. To investigate whether glucose availability influences oxidative stress following iRFA, intracellular ROS levels were dynamically monitored using the DCFH-DA fluorescent probe under different glucose conditions after 46 °C thermal exposure.

**Figure 3.**
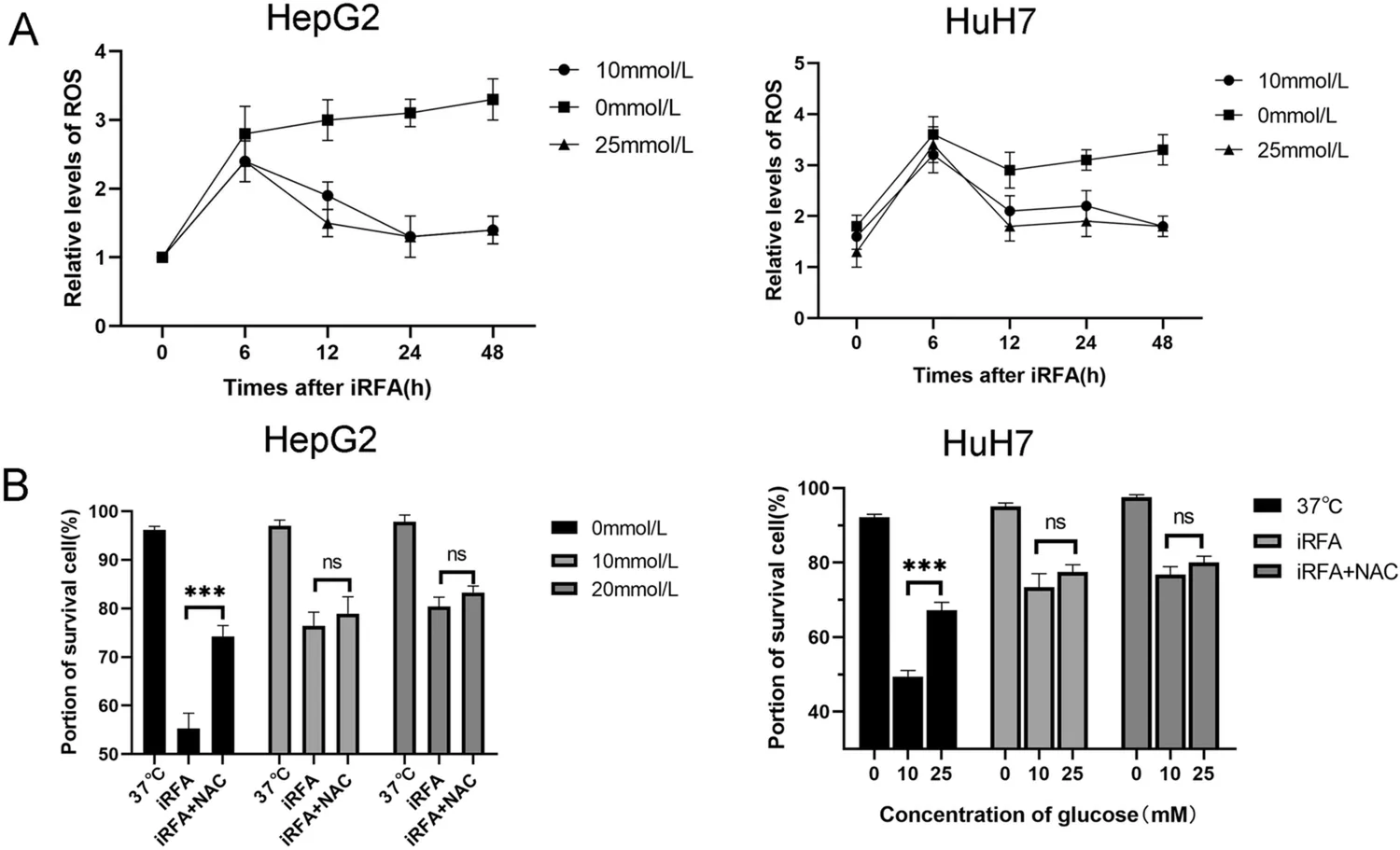
Increased glucose uptake after iRFA is associated with dynamic changes in ROS levels. (A) Intracellular ROS levels were measured in HepG2 and Huh7 cells cultured in media containing 0, 10, or 25 mM glucose at the indicated time points following iRFA. (B) Cell death was assessed after treatment with the ROS scavenger *N*-acetylcysteine (NAC, 5 mM) administered 12 h after iRFA. *p < 0.05; **p < 0.01; ***p < 0.001. Data are presented as mean ± SD from three independent experiments.

As shown in Figure 3A, iRFA induced distinct temporal patterns of ROS accumulation depending on extracellular glucose availability. In cells cultured in 10 mM or 25 mM glucose, intracellular ROS levels increased rapidly after iRFA, reached a peak at approximately 6 h, and subsequently declined toward baseline levels. In contrast, cells cultured under glucose-deprived conditions (0 mM glucose) exhibited a sustained increase in ROS levels throughout the observation period(Figure 3A), indicating impaired control of oxidative stress following thermal injury.

To further examine the contribution of ROS accumulation to cell fate after iRFA, the ROS scavenger NAC (5 mM) was administered 12 h after thermal treatment. NAC treatment significantly improved cell viability and reduced cell death in the 0 mM glucose group (Figure 3B). By contrast, NAC had minimal effects on cell viability or cell death in the 10 mM and 25 mM glucose groups.

Taken together, these findings suggest that extracellular glucose availability is closely associated with the regulation of intracellular ROS levels following iRFA. Enhanced glucose uptake may facilitate the maintenance of redox homeostasis and thereby contribute to the survival of HCC cells exposed to sublethal thermal stress.

### GLUT4 contributes to iRFA-induced glucose uptake adaptation

**Figure 4.**
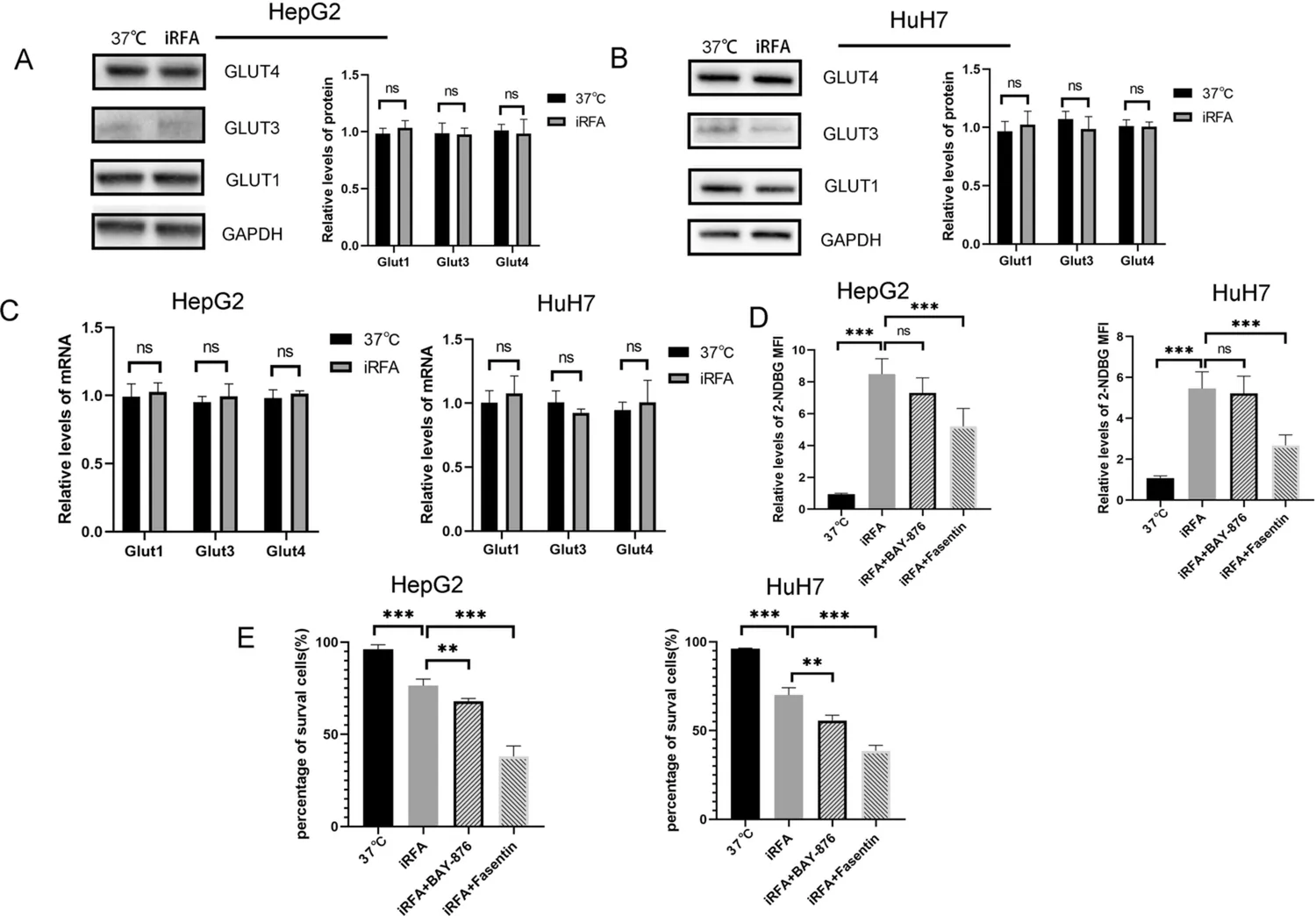
GLUT4 is a key mediator of iRFA-induced glucose uptake. (A, B, C) Protein (A,B) and mRNA (C) expression levels of GLUT1, GLUT3, and GLUT4 were analyzed in HepG2 and Huh7 cells 24 h after iRFA. (D) Glucose uptake was measured after treatment with the GLUT1 inhibitor BAY-876 or the GLUT4 inhibitor Fasentin following iRFA. (E) Apoptosis of HCC cells treated with BAY-876 or Fasentin was assessed after iRFA. *p < 0.05; **p < 0.01; ***p < 0.001. Data are presented as mean ± SD from three independent experiments.

To identify the glucose transporter responsible for the enhanced glucose uptake following iRFA, we examined the expression profiles of major glucose transporter family members, including GLUT1, GLUT3, and GLUT4, in HCC cells. PCR and Western blot analyzes performed at 24 h after iRFA showed that the mRNA and total protein levels of GLUT1 and GLUT4 remained largely unchanged following thermal exposure in both HepG2 and HuH7 cells (Figures 4A–C). In addition, GLUT3 expression was relatively low in both cell lines, suggesting that GLUT3 is unlikely to be a major contributor to the increased glucose uptake after iRFA.

Because total transporter expression was not significantly altered, we hypothesized that functional regulation of glucose transporters, rather than transcriptional upregulation, may contribute to the enhanced glucose uptake following iRFA. To further investigate the functional contribution of different glucose transporters, pharmacological inhibition experiments were performed following iRFA treatment. Inhibition of GLUT1 using BAY-876 did not significantly affect iRFA-induced glucose uptake, although it resulted in a mild increase in cell death following iRFA treatment (Figure 4D and 4E). In contrast, inhibition of GLUT4 using fasentin markedly reduced iRFA-induced glucose uptake and significantly increased cell death after thermal stress (Figure 4D and 4E). These results indicate that GLUT4 plays a predominant role in mediating the adaptive increase in glucose uptake following iRFA and contributes to the survival of HCC cells under sublethal thermal stress.

Given that total GLUT4 expression remained unchanged after iRFA, these findings suggest that GLUT4 contributes to glucose uptake adaptation through functional regulation, potentially involving post-translational activation or altered subcellular localization rather than increased protein expression.

### iRFA induces GLUT4 translocation to the plasma membrane and promotes HCC cell survival

GLUT4 can redistribute from intracellular storage compartments to the plasma membrane in response to metabolic stimuli, such as insulin stimulation. Therefore, we investigated whether iRFA induces GLUT4 translocation as an adaptive response to sublethal thermal stress.

**Figure 5.**
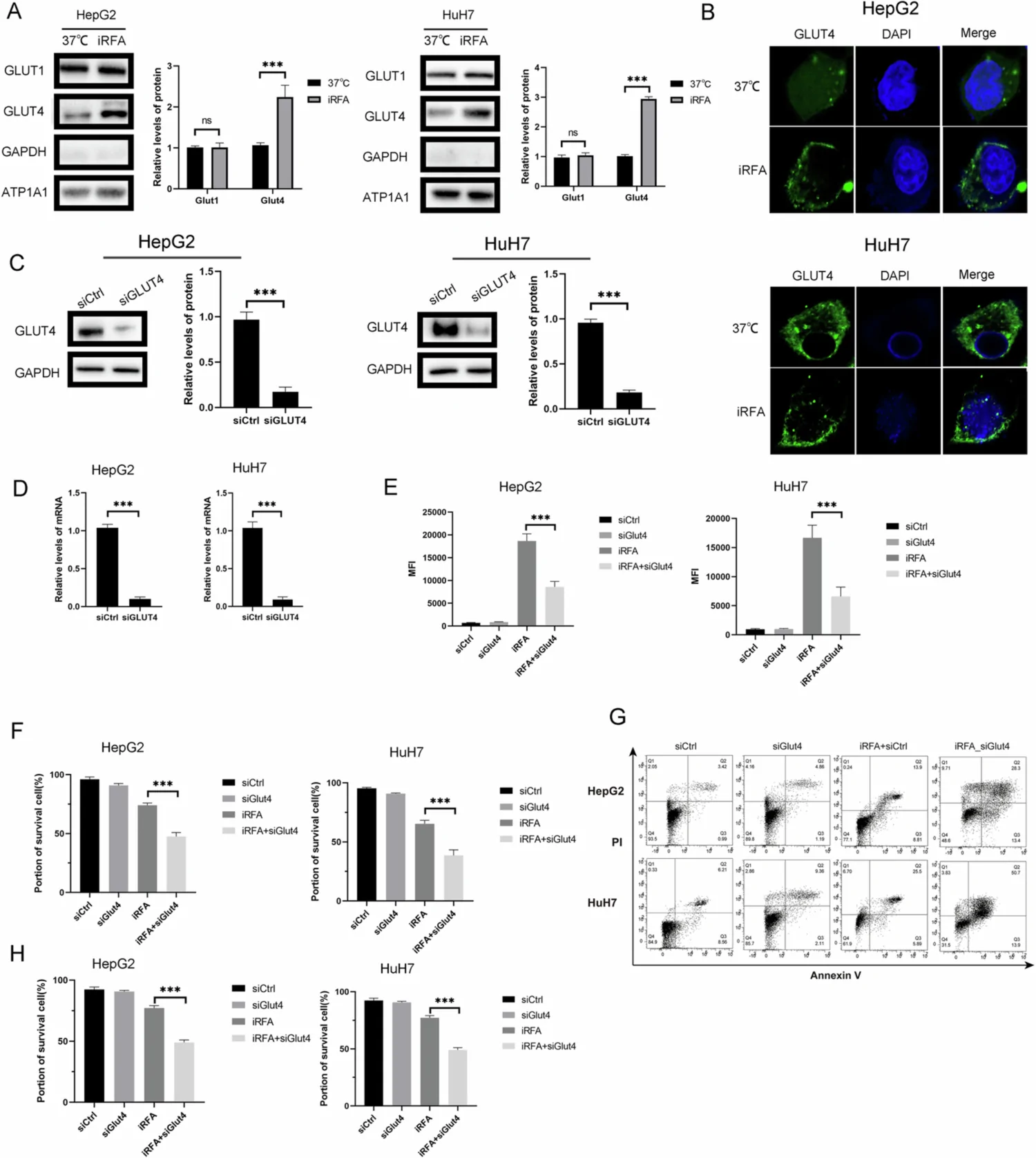
iRFA induces GLUT4 translocation to the plasma membrane. (A) Membrane-associated GLUT4 levels were analyzed in HepG2 and Huh7 cells 24 h after iRFA. (B) Immunofluorescence staining showing redistribution of GLUT4 to the plasma membrane following iRFA. (C, D) Efficiency of GLUT4 knockdown by siRNA was confirmed at the protein (C) and mRNA (D) levels. (E) Glucose uptake was measured in control and GLUT4-silenced cells under basal conditions and after iRFA. (F) Cell death was assessed in control and GLUT4 knockdown cells following iRFA. (G) Cell death in control and GLUT4 knockdown cells was quantified using Annexin V-FITC/PI staining and flow cytometry after iRFA. (H) The statistical results of G in three independent experiments. *p < 0.05; **p < 0.01; ***p < 0.001. Data are presented as mean ± SD from three independent experiments.

Analysis of membrane-associated proteins revealed that GLUT4 abundance at the plasma membrane was significantly increased at 24 h after iRFA treatment in both HepG2 and HuH7 cells (Figure 5A). Consistently, immunofluorescence staining demonstrated enhanced localization of GLUT4 at the plasma membrane following iRFA exposure (Figure 5B). These findings indicate that iRFA promotes functional redistribution of GLUT4 rather than transcriptional or translational upregulation.

To further determine the functional role of GLUT4 in glucose uptake adaptation after iRFA, GLUT4 expression was silenced using specific siRNA. Efficient knockdown of GLUT4 was confirmed by quantitative PCR and Western blot analysis (Figure 5C and 5D). GLUT4 depletion had limited effects on glucose uptake and cell viability under basal conditions. However, following iRFA exposure, GLUT4 knockdown significantly reduced glucose uptake compared with siControl cells, indicating that GLUT4 is required for the enhanced glucose uptake response induced by thermal stress (Figure 5E).

Furthermore, CCK-8 assays demonstrated that GLUT4 silencing significantly decreased cell viability after iRFA(Figure 5F). Consistently, Annexin V/PI flow cytometric analysis showed that siGLUT4-transfected cells exhibited a markedly increased proportion of cell death compared with siControl cells following iRFA treatment (Figure 5G and 5H). These findings demonstrate that GLUT4-mediated glucose uptake contributes to the survival adaptation of HCC cells after incomplete ablation.

## Discussion

Our study demonstrates that glucose uptake is significantly enhanced by iRFA in hepatocellular carcinoma cells, which is associated with reduced intracellular ROS levels and improved cell survival during iRFA. Mechanistically, the upregulated glucose uptake is mediated by the translocation of GLUT4 from the cytoplasm to the plasma membrane, rather than changes in total GLUT4 expression.

iRFA inevitably exposes HCC cells to a transient, sublethal heat stimulus that can profoundly remodel cellular metabolic states. It has been reported that thermal stress markedly enhances glucose uptake and induces metabolic reprogramming, with alterations in glucose metabolism being particularly prominent.^11–13,15^ In tumor cells, increased glucose uptake is among the earliest and most prominent metabolic alterations and is closely associated with tumor initiation, progression, and malignant phenotypes. This metabolic shift is thought to reflect an adaptive response to the elevated energetic and biosynthetic demands of rapid proliferation, which is commonly achieved through the upregulation of glucose transporters.^16–18^ Enhanced glucose uptake not only supplies ATP but also provides essential intermediates for nucleotide, lipid, and amino acid synthesis, thereby supporting cell cycle progression and tumor growth. In HCC and other solid malignancies, glucose metabolic reprogramming correlates with tumor differentiation, aggressiveness, and adverse outcomes.^19^ Clinically, the high glucose avidity of tumors underlies the molecular basis of ^18^F-FDG PET/CT imaging, widely used for diagnosis, treatment evaluation, and recurrence monitoring.^20^ Our study demonstrates that iRFA significantly enhances glucose uptake in HCC cells, which contributes to the suppression of intracellular ROS levels and promotes cell survival. Heat stress mediated upregulation of glucose uptake may represent a critical mechanism underlying metabolic activation and malignant progression of residual tumor cells. Inhibition of glucose uptake or the activity of key glycolytic enzymes has been shown to significantly reduce tumor cell viability and enhance sensitivity to thermal, chemotherapeutic, or radiotherapeutic stress.^21–24^ Thus, glucose uptake not only serves as a hallmark of tumor metabolic reprogramming but also directly influences cell survival under stress conditions.

Moreover, our data indicate that thermal stress, as a non-canonical physiological stimulus, can similarly trigger GLUT4 membrane translocation. GLUT4 is a key facilitative glucose transporter responsible for mediating glucose uptake from the extracellular space into cells in various cell types.^25^ Evidence from various cell types indicates that impairment of GLUT4 function or inhibition of its membrane translocation reduces glucose uptake and NADPH production, leading to ROS accumulation and decreased cell survival.^26–28^ Under basal conditions, GLUT4 is primarily sequestered intracellularly, translocating to the plasma membrane only in response to specific stimuli, such as insulin or cellular stress.^25^ This mechanism resembles observations in cardiomyocytes, where oxidative stress induces GLUT4 translocation via the PI3K/Akt and AMPK signaling axes, dynamically regulating transporter distribution in response to intracellular and extracellular environmental changes.^29–31^ In our context, the redistribution of GLUT4 to the plasma membrane under thermal stress may represent a rapid adaptive strategy to increase glucose uptake, allowing tumor cells to swiftly acquire glucose for energy production and biosynthesis following heat-induced damage. This observation aligns with the Warburg effect commonly seen in cancer cells, whereby glucose uptake and glycolysis remain high even under aerobic conditions to meet energy and metabolic demands.

Several limitations of this study should be acknowledged. First, all experiments were performed in vitro, which cannot fully recapitulate the complex biological processes occurring in vivo following RFA. The sublethal heat-treatment model (46 °C for 15 min) was designed to mimic residual tumor cells after insufficient RFA; however, it does not reproduce the heterogeneous thermal distribution that exists within tumors during clinical RFA procedures. Second, important components of the tumor microenvironment, including vascular perfusion, immune-cell infiltration, inflammatory responses, and nutrient availability, were not represented in our experimental system. These factors may substantially influence tumor cell behavior and glucose metabolism after iRFA. Third, tumor heterogeneity among different cellular subpopulations was not fully addressed in the present study. Therefore, the biological effects observed in cultured cells may not completely reflect responses in clinical tumors. Future studies using animal models and clinical specimens are warranted to validate the current findings and further elucidate the role of GLUT4 in residual tumor progression following iRFA.

## Conclusions

Collectively, the iRFA–GLUT4 membrane translocation–enhanced glucose uptake axis constitutes a potential metabolic adaptation pathway that enables residual tumor cells to survive under energetic stress and adverse microenvironmental conditions, increasing the risk of recurrence. This mechanism not only provides a metabolic perspective on HCC cell survival post-iRFA but also highlights stress-induced GLUT4 dynamics as a potential therapeutic target. Further investigation into the direct relationship between GLUT4 translocation and tumor resistance may facilitate the development of more precise combination therapies.

## Data Availability

The raw data supporting the conclusions of this article will be made available by the authors, without undue reservation.
